# Associations between circulating microRNAs and lipid-rich coronary plaques measured with near-infrared spectroscopy

**DOI:** 10.1038/s41598-023-34642-6

**Published:** 2023-05-10

**Authors:** Julie Caroline Sæther, Elisabeth Kleivhaug Vesterbekkmo, Maria Dalen Taraldsen, Bruna Gigante, Turid Follestad, Helge Rørvik Røsjø, Torbjørn Omland, Rune Wiseth, Erik Madssen, Anja Bye

**Affiliations:** 1 Department of Circulation and Medical Imaging, Norwegian University of Science and Technology, Trondheim, Norway; 2 Department of Cardiology, St. Olavs Hospital, Trondheim, Norway; 3 National Advisory Unit on Exercise Training as Medicine for Cardiopulmonary Conditions, Trondheim, Norway; 4 Division of Cardiovascular Medicine, Karolinska Institutet, Stockholm, Sweden; 5 Department of Clinical and Molecular Medicine, Norwegian University of Science and Technology, Trondheim, Norway; 6 Clinical Research Unit Central Norway, St. Olavs Hospital, Trondheim, Norway; 7 Division of Research and Innovation, Akershus University Hospital, Lørenskog, Norway; 8 K. G. Jebsen Center for Cardiac Biomarkers, University of Oslo, Oslo, Norway; 9 Department of Cardiology, Division of Medicine, Akershus University Hospital, Lørenskog, Norway

**Keywords:** Biomarkers, Cardiology, Interventional cardiology

## Abstract

Lipid-rich coronary atherosclerotic plaques often cause myocardial infarction (MI), and circulating biomarkers that reflect lipid content may predict risk of MI. We investigated the association between circulating microRNAs (miRs) are lipid-rich coronary plaques in 47 statin-treated patients (44 males) with stable coronary artery disease undergoing percutaneous coronary intervention. We assessed lipid content in non-culprit coronary artery lesions with near-infrared spectroscopy and selected the 4 mm segment with the highest measured lipid core burden index (maxLCBI_4mm_). Lipid-rich plaques were predefined as a lesion with maxLCBI_4mm_ ≥ 324.7. We analyzed 177 circulating miRs with quantitative polymerase chain reaction in plasma samples. The associations between miRs and lipid-rich plaques were analyzed with elastic net. miR-133b was the miR most strongly associated with lipid-rich coronary plaques, with an estimated 18% increase in odds of lipid-rich plaques per unit increase in miR-133b. Assessing the uncertainty by bootstrapping, miR-133b was present in 82.6% of the resampled dataset. Inclusion of established cardiovascular risk factors did not attenuate the association. No evidence was found for an association between the other analyzed miRs and lipid-rich coronary plaques. Even though the evidence for an association was modest, miR-133b could be a potential biomarker of vulnerable coronary plaques and risk of future MI. However, the prognostic value and clinical relevance of miR-133b needs to be assessed in larger cohorts.

## Introduction

Coronary atherosclerosis complicated with plaque rupture or erosion often cause myocardial infarction (MI)^1^. It has been demonstrated that a high lipid content in coronary atherosclerotic lesions increases the risk of cardiovascular events, such as MI^2–6^. For instance, Erlinge et al.^6^ demonstrated that patients with one or more untreated lipid-rich coronary artery lesions, defined as a plaque with maximum lipid core burden index within any 4 mm segment length across the entire lesion (maxLCBI_4mm_) of ≥ 324.7, were at increased risk of non-culprit lesion-related major adverse cardiac events. Near infrared spectroscopy (NIRS) is one intracoronary imaging method that can identify lipid content in coronary atherosclerotic lesions^7^, but its application and invasive approach is not established in contemporary clinical practice. It is therefore of clinical interest to identify non-invasive biomarkers that reflects lipid content in coronary plaques.

Circulating microRNAs (miRs) represents promising biomarkers of vulnerable coronary plaque characteristics and the associated risk of MI. miRs are small endogenous non-coding RNAs that regulates post-transcriptional gene expression of nearly 60% of the human protein-coding genes^8^. They are essential mediators of many molecular pathways and functions and known to be involved in most processes related to the pathology of coronary atherosclerosis, contributing to plaque formation, progression, rupture, and erosion^9^. To date, several circulating miRs have been suggested as potential prognostic and diagnostic biomarkers of both acute- and chronic cardiovascular disease (CVD)^10–12^. Still, few studies have investigated the association between circulating miRs and vulnerable coronary plaque characteristics assessed with advanced imaging techniques^13–18^. Among those performed, none have assessed lipid content using NIRS, and the studies are limited by the inclusion of few predefined miRs. Thus, in the present study, we aimed to investigate whether circulating miRs in plasma are associated with lipid-rich coronary plaques, measured as maxLCBI_4mm_ ≥ 324.7 by NIRS, in statin-treated patients with stable coronary artery disease (CAD).

## Methods

### Study design and patients

This post-hoc study used baseline data from the randomized controlled trial *Impact of Cardiac Exercise Training on Lipid Content in Coronary Atheromatous Plaques Evaluated by Near-Infrared Spectroscopy* (CENIT)^19^. The trial was registered at clinicaltrials.gov (NCT02494947), approved by the ethical committee of central Norway (2015/210), and performed in accordance with the Declaration of Helsinki. Patients who underwent coronary angiography at St. Olavs Hospital in Trondheim, Norway, 2016–2019, and were diagnosed with a hemodynamic significant coronary artery stenosis in one or more epicardial vessels requiring percutaneous coronary intervention (PCI) were screened for inclusion. To be eligible for inclusion, patients had to be on stable statin therapy for ≥ 6 weeks before intracoronary imaging and have no previous coronary artery bypass surgery or inflammatory diseases other than atherosclerosis. Written informed consent were obtained from 60 patients.

### Intracoronary imaging

**Figure 1:**
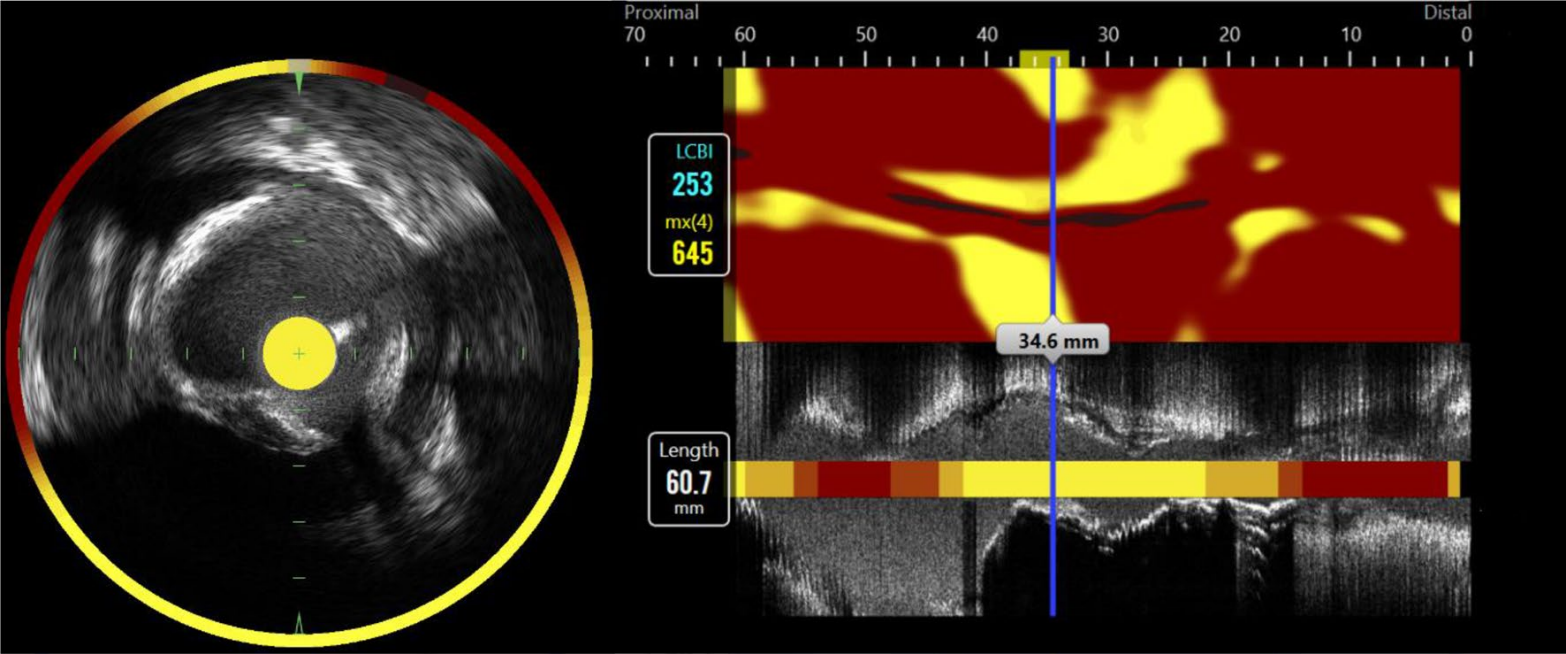
Plaque lipid content in the circumflex artery measured by near infrared spectroscopy. To the left: Cross-section image with surrounding color-coded pixels representing lipid accumulation within the plaques. To the right: Chemogram demonstrating the most severe lesion with maxLCBI_4mm_ of 645. Color-code pixels spans from red to yellow with increasing probability of lipid. *maxLCBI*_*4mm*_ maximum lipid core burden index within any 4 mm segment across the entire lesion.

Following successful drug-eluting stent implantation and administration of intracoronary nitroglycerine (200 µg), three-vessel imaging by near infrared spectroscopy intravascular ultrasound (NIRS-IVUS) was performed when feasible in non-culprit coronary lesions. The combined NIRS-IVUS catheter (TVC-MC8 model system with a 3.2Fr 40 MHz catheter, Infraredx, Burlington, Massachusetts) was positioned as distal as possible in the coronary artery and automatically pulled back at a fixed rate of 0.5 mm/s. The commercial software Pie Medical Imaging Software (CAAS Intravascular) was used to analyze anonymized angiograms and intracoronary imaging data by an independent core facility (KCRI, Krakow, Poland). NIRS generates chemograms with color-coded pixels, spanning from red to yellow, illustrated the probability of lipid-rich plaques, with yellow pixels representing the highest probability (Fig. 1). This allowed for calculation of the lipid core burden index (LCBI) that ranges from 0 to 1000, equivalent to the percentage of yellow pixels^20^. The present study selected the coronary artery segment with the highest measured lipid content, defined as maxLCBI_4mm_, as target segment. Patients were further divided into two predefined groups based on their maxLCBI_4mm_ values: maxLCBI_4mm_ < 324.7 and maxLCBI_4mm_ ≥ 324.7, where maxLCBI_4mm_ ≥ 324.7 represents coronary plaques with high lipid content, predefined as lipid-rich plaques, with an increased risk of future coronary events^6^.

### Variable collection

In the morning, the day after the intracoronary imaging procedures, fasting venous blood samples were collected in 5 mL ethylenediamine tetraacetic acid tubes, centrifuged for 20 min in 20 °C at 3000×*g* (Rotina 420R, Hettich zentrifugen), aliquoted and stored in − 80 °C freezer until miR analyses. Additional blood samples were analyzed for total cholesterol, total triglycerides, LDL cholesterol (LDL-C), HDL cholesterol (HDL-C), lipoprotein (a), Apolipoprotein-B, Apolipoprotein-A1, glycated hemoglobin A1c, hemoglobin, and creatinine by standard in-hospital procedures at the Department of Medical Biochemistry, St. Olavs Hospital. At the time of inclusion, information on anthropometry and CVD risk factors were collected from the hospital medical records. This included sex, age, body mass index (kg·m^−2^), diabetes mellitus, systolic- and diastolic blood pressure, smoking status, other comorbidities, medications, previous CVD (CAD, stroke, peripheral arterial disease, and/or aortic disease), heredity for CVD [first-degree relative with CVD before the age of 55 years (father) and 65 years (mother)], hyperlipidemia, and medically treated hypertension. The two latter conditions were defined as previously being diagnosed with this condition or not by a general practitioner or in an outpatient clinic before enrollment in the present study.

### Sample preparation and real-time quantitative polymerase chain reaction

Frozen plasma samples were sent to Qiagen Genomic Services (Hilden, Germany) for miR isolation and quantification. The plasma samples were thawed on ice and centrifuged in a 4 °C microcentrifuge for 5 min at 3000×*g*. An aliquot of 200 μL was further transferred to a FluidX tube and mixed for 1 min with 60 μL of Lysis solution BF (containing RNA spike-in template mixture and 1 μg carrier-RNA per 60 μL Lysis Solution BF) before incubation for 7 min at room temperature. Furthermore, 20 μL Protein Precipitation solution BF were added to the samples. The miRCURY RNA isolation Kit (Biofuids; high-throughput bead-based protocol v.1, Hilden, Germany) were used to extract RNA from the samples in an automated 96 well format, and purified total RNA was eluted in 50 μL. Pre-mixed RNA spike-ins, including UniSp2, UniSp4, and UniSp5, were added to the purification as RNA extraction controls, to detect differences in extraction efficiency. In later steps, UniSp3 was added to control for inhibition at the quantitative polymerase chain reaction (qPCR) level.

A total of 7 μL RNA was reverse transcribed (RT) to complementary DNA (cDNA) in 35 μL reactions using the miRCURY LNA RT Kit (QIAGEN). The pre-mixed spike-in, UniSp6, was added in the RT step as a cDNA synthesis control to confirmed that the RT and amplification occurs with equal efficiency in all samples. cDNA was diluted 50 times and assayed in 10 μL PCR reactions using the miR Ready-to-Use PCR, Serum/Plasma Focus panel with miRCURY LNA SYBR Green master mix. Each miR was assayed once by qPCR. 177 miRs were included in the predefined Serum/Plasma Focus panel and analyzed in the samples. A “no template” sample (negative control) was included in the RT step and profiled like the samples to detect potential RNA contamination in the RT step.

The amplification was performed in a LightCycler® 480 Real-Time PCR System (Roche) in 384 well plates. The amplification curves were analyzed using the Roche LC software, both for determination of quantification cycles (Cq), by the 2nd derivative method, and for melting curve analysis. All assays were inspected for distinct melting curves and the melting temperature was checked to be within known specifications for the assay. The Cq value of all assays were compared to background level from the negative control sample and had to be detected with 5 Cq less than the negative control. Quality control inspection of the samples’ spike-in raw Cq-values, hemolysis values, average Cq value for the expressed miRs, and the principal component analysis were performed in collaboration with Qiagen Genomic Services to detect deviated samples unsuitable for statistical analyses. All data was normalized to the average of assays detected in all samples (global mean), as this was detected as the most stable normalizer of our data (NormFinder software)^21^. The formula for calculating normalized Cq was: *Global mean Cq (sample 1)– assay Cq (miR of interest in sample 1), global mean Cq (sample 2)– assay Cq (miR of interest in sample 2), and so on for all included samples.*

### Statistical analyses

IBM SPSS Statistics (version 27.0, Armonk, NY: IBM Corp) and R^22^ were used to analyze the data. Continuous data are presented as mean with standard deviation and categorical data as frequencies with percentages. miRs present in ≥ 80% of the study population were included in the statistical analyses. The lowest measured concentration in the specific assay was used for miR concentration below the level of detection. Shapiro–Wilk tests and QQ-plots were used to evaluate normal distribution of miRs and continuous patient characteristics data. Univariable analyses for the association between concentrations of each miR and dichotomized maxLCBI_4mm_ (maxLCBI_4mm_ < 324.7 or ≥ 324.7) were performed by independent sample *t* tests or Mann Whitney U-tests as appropriate. Adjusted *p* values were calculated using the Benjamini–Hochberg method, controlling the false discovery rate at 0.05. Patient characteristics were compared between the groups by independent sample *t* tests, Mann Whitney U tests, or Chi-square test as appropriate, and *P* < 0.05 were considered statistically significant.

Penalized logistic regression by the elastic net method, implemented in the *glmnet* package in R^23^, was used for multivariable analyses. Elastic net performs parameter estimation and model selection simultaneously. The complexity of the model is reduced by imposing a penalty, such that the regression coefficients for variables with low predictive value are shrunken towards zero (odds ratio (OR) = 1), or to exactly zero for some variables. Elastic net is a combination of least absolute shrinkage and selection operator (lasso) and ridge regression [as specified by a parameter α (0.1)]. A model closer to lasso than ridge (α = 0.9) was used. The degree of shrinkage was determined by tenfold cross-validation. The tenfold cross-validation was repeated ten times to reduce the effect of randomness in the selection of the ten folds, and the penalty was selected to minimize the average of the ten mean deviances. The uncertainty of the estimated OR from the elastic net was assessed by bootstrapping. The fitting procedure was repeated for 1000 bootstrap samples, and the uncertainty for each variable was represented by the proportion of the bootstrap samples for which its coefficient was not set to zero (OR not set to 1) in the estimated model. Models for two sets of predictors were estimated: one model including miRs only (N = 160), and one model including both miRs and established risk factors for CVD (n = 174). The CVD risk factors included age, body mass index, smoking, medically treated hypertension, diabetes mellitus, hyperlipidemia, heredity of CVD, previous CVD, total cholesterol, LDL-C, HDL-C, total triglycerides, LDL-C/HDL-C, and lipoprotein (a). For each model, the top ten variables, based on the percent times inclusion in the 1000 bootstrap samples, are presented in the results section. The remaining results are included in Supplementary Information.

The Spearman correlation were used to assess the dependence between the miRs presented in the result section. A heatmap was computed by GraphPad Prism version 9. Mann Whitney U tests and Spearman correlations were used to investigate the associations between selected miRs and patient characteristics and risk factors for CVD. *P* < 0.05 were considered statistically significant. Receiver operating characteristic (ROC) curve analyses were performed to assess the predictive performance of the selected miR. The ROC curve and the area under the curve (AUC) were calculated by cross-validation based on a logistic regression model with the selected miR as the only variable, and for a model with traditional lipid measurements. Traditional lipid measurements included LDL-C, HDL-C, triglycerides, LDL/HDL, and lipoprotein (a).

## Results

From the 60 included patents in the CENIT trial^19^, 13 patients were excluded from the present study due to non-evaluable NIRS data (no non-culprit plaques), missing blood samples, or disregarded plasma samples based on quality control tests (Supplementary Figure S1). This yielded 47 eligible patients for statistical analyses. Out of the 177 analyzed miRs, 160 miRs had detectable concentrations in ≥ 80% of the patients and was included in the statistical analyses. There were no significant differences in patient characteristics between patients with and without lipid-rich plaques (*P* < 0.05, Table 1). The mean (standard deviation) of maxLCBI_4mm_ for those with and without lipid-rich plaques are 427.0 (85.8) and 153.0 (91.0), respectively, and the corresponding values for total LCBI are 194.0 (75.6) and 52.7 (42.3) (Table 2). The distribution of maxLCBI_4mm_ measurements in both groups are illustrated in Supplementary Figure S2).

**Table 1:** Patient characteristics of the study population (N = 47).

	maxLCBI_4mm_ < 324.7 N = 24	maxLCBI_4mm_ ≥ 324.7 N = 23	*P* value
General			
Age, years	58 ± 8.3	58 ± 6.4	0.88
Males, (n, %)	21 (87.5%)	23 (100%)	0.08
Body mass index, kg m^−2^	28.8 ± 3.1	28.8 ± 4.2	0.67
Current smoker or ex-smoker (n, %)	16 (66.7%)	14 (60.9%)	0.68
Systolic blood pressure, mmHg	143.0 ± 16.6	145.1 ± 17.1	0.67
Diastolic blood pressure, mmHg	82.7 ± 7.9	85.0 ± 8.5	0.33
Medical history, previously diagnosed			
Diabetes mellitus (n, %)	3 (12.5%)	2 (8.7%)	0.67
Medically treated hypertension (n, %)	15 (62.5%)	10 (43.5%)	0.19
Hyperlipidemia (n, %)	8 (33.3%)	7 (30.4%)	0.83
Heredity of premature CVD (n, %)	19 (79.2%)	21 (95.5%)	0.10
Prior history of CVD (n, %)	10 (41.7%)	13 (56.5%)	0.31
Medication			
Statins (n, %)	24 (100%)	23 (100%)	1.00
Dual antiplatelet therapy (n, %)	21 (87.5%)	21 (91.3%)	0.67
Combined therapy with ezetimibe (n, %)	1 (4.2%)	3 (13.0%)	0.28
Calcium blockers (n, %)	5 (20.8%)	3 (13.0%)	0.48
Beta-blockers (n, %)	12 (50.0%)	7 (30.4%)	0.17
ACE inhibitors/angiotensin II receptor antagonists (n, %)	16 (66.7%)	10 (43.5%)	0.11
Diuretics (n, %)	3 (12.5%)	2 (8.7%)	0.67
Clinical measurements			
Total cholesterol, mmol/L	3.8 ± 0.7	3.8 ± 1.0	0.57
LDL cholesterol, mmol/L	2.2 ± 0.6	2.2 ± 0.9	0.35
HDL, mmol/L	1.0 ± 0.3	1.0 ± 0.2	0.69
Triglycerides, mmol/L	1.5 ± 0.8	1.5 ± 0.7	0.89
Apolipoprotein A1, g/L	1.3 ± 0.2	1.2 ± 0.1	0.45
Apolipoprotein B, g/L	0.8 ± 0.2	0.7 ± 0.3	0.22
Lipoprotein a, mg/L	369.3 ± 421.5	534.3 ± 575.2	0.15
Creatinine, µmol/L	79.7 ± 14.4	78.9 ± 12.3	0.92
Hemoglobin, g/dL	14.4 ± 1.4	14.2 ± 2.0	0.81
Glycosylated hemoglobin, %	5.7 ± 1.1	5.5 ± 1.4	0.34

Data are presented as mean ± standard deviation or numbers with percentages.

*maxLCBI*_*4mm*_ the maximum lipid core burden index within any 4 mm segment across the entire lesion, *CVD* cardiovascular disease, *ACE* angiotensin-converting-enzyme, *LDL* low-density lipoprotein, *HDL* high-density lipoprotein.

**Table 2:** NIRS-IVUS derived lesion- and plaque characteristics in target segments.

	maxLCBI_4mm_ < 324.7 N = 24	maxLCBI_4mm_ ≥ 324.7 N = 23	*P* value
Target segment			
Left anterior descending artery (n, %)	11 (45.8%)	8 (34.8%)	0.44
Circumflex artery (n, %)	4 (16.7%)	5 (21.7%)	0.67
Right coronary artery (n, %)	9 (37.5%)	10 (43.5%)	0.68
NIRS-IVUS derived lesion- and plaque characteristics			
Plaque burden, %	46.7 ± 8.6	51.2 ± 7.3	0.06
Minimal lumen area, mm^2^	5.3 ± 1.8	4.7 ± 2.2	0.06
Stenosis at minimal lumen area, %	60.9 ± 11.5	66.7 ± 11.0	0.10
Plaque volume, mm^3^	157.8 ± 99.2	215.7 ± 122.8	0.11
Vessel volume, mm^3^	326.78 ± 193.7	429.9 ± 261.5	0.17
Lumen volume, mm^3^	169.0 ± 99.7	214.2 ± 146.4	0.39
Segment length, mm	21.3 ± 11.9	27.5 ± 14.2	0.15

Data are presented as numbers with percentages or mean ± standard deviation.

*NIRS-IVUS* near-infrared spectroscopy intravascular ultrasound, *maxLCBI*_*4mm*_ the maximum lipid core burden index within any 4 mm segment across the entire lesion, *LCBI* lipid core burden index.

From the univariable analyses comparing miR concentrations between patients with and without a lipid-rich lesion, miR-miR-18a-5p (*P* = 0.008), miR-133b (*P* = 0.003), 15a-5p (*P* = 0.030), miR-320c (*P* = 0.042), and miR-423-5p (*P* = 0.047) were the five miRs with the smallest *P* values. However, none of the comparisons were statistically significant after adjustment for multiple testing. Two statistical models with two sets of predictors were estimated in the multivariable analyses. This included one model with miRs only and one model with both miRs and established risk factors for CVD. In the miRs only model, miR-133b was the miR most strongly associated with lipid-rich coronary plaques in patients with stable CAD according to the percentage presence in the resampled dataset by bootstrapping (82.6%). The estimated OR for miR-133b was 1.18, which indicate that the odds of lipid-rich plaques increase with 18% for every unit increase in miR-133b. In the model with miRs and established CVD risk factors, miR-133b remained as the variable most strongly associated with coronary lipid-rich plaques with 84.2% presence in the resampled dataset and an OR of 1.15. The top ten miRs and the top ten miRs and CVD risk factors for the two sets of predictors are presented in Table 3, with miR-133b as the only selected variable in both models. Additional results are included in Supplementary Table S1, and a heatmap illustrating the dependence between miRs presented in Table 3 is shown in Supplementary Figure S3. miR-133b were not found to be associated with patient characteristics or risk factors for CVD (results not shown). From the ROC analyses, the cross-validated AUC for a logistic regression model with miR-133b as the only variable was 0.70 (95% confidence interval from 0.55 to 0.84), which emphasizes the potential predictive value of miR-133b (Supplementary Figure S4). For comparison, the similar values for a logistic regression model including traditional lipid measurements (LDL-C, HDL-C, total triglycerides, lipoprotein (a) and LDL/HDL) was 0.67 (95% confidence interval from 0.51 to 0.81).

**Table 3:** Estimated odds ratios for lipid-rich plaques (maxLCBI_4mm_ ≥ 324.7) from elastic net of the top ten miRs and the top ten miRs and CVD risk factors, according to the percentage presence in the 1000 bootstrap samples.

miRs only	Estimated odds ratio	Percent presence in samples from bootstrap
miR-133b	1.18	82.6
miR-18a-5p	1	35.6
miR-100-5p	1	35.0
miR-320d	1	32.8
miR-7-5p	1	31.2
miR-130a-3p	1	29.0
miR-22-5p	1	28.4
miR-590-5p	1	28.2
miR-222-3p	1	27.5
miR-15a-5p	1	26.5
miRs and established CVD risk factors		
miR-133b	1.15	84.2
Hereditary CVD	1	54.9
miR-18a-5p	1	33.3
miR-320d	1	32.8
miR-100-5p	1	31.9
miR-7-5p	1	31.5
miR-130a-3p	1	31.0
miR-22-5p	1	29.3
miR-15b-3p	1	28.7
miR-590-5p	1	27.6

*miR/miRs* microRNA(s), *3p* 3' arm of the precursor duplex, *5p* 5' arm of the precursor duplex, *maxLCBI*_*4mm*_ the maximum lipid core burden index within any 4 mm segment across the entire lesion, *CVD* cardiovascular disease.

## Discussion

In the present study, we investigated the association between 160 plasma miRs and lipid-rich coronary plaques, measured with NIRS, in patients with stable CAD. Our study demonstrated that (1) miR-133b was the miR most strongly associated with lipid-rich coronary plaques, and (2) the association between miR-133b and lipid-rich coronary plaques was unaffected by the inclusion of established CVD risk factors.

Coronary atherosclerosis is a chronic inflammatory vascular disorder with a multifactorial origin that initiates endothelial injury and thereby causes subendothelial infiltration and accumulation of lipoproteins and immune cells, such as macrophages, and subsequent formation of coronary atherosclerotic lesions^24^. Macrophages play a significant role in the atherosclerotic process and plaque formation and stabilization by initiating and sustaining local inflammation that promotes lipoprotein retention^25^. miR-133b, which is coded by the *MIR133b* gene and located on chromosome 6p12.2, is known to be expressed in the skeletal muscles and to play essential roles in macrophage functions and processes related to muscle development, muscle cell metabolism, and homeostasis^26^. miR-133b is also found to be involved in many pathways related to the pathogenesis of coronary atherosclerosis, such as the Notch-signaling pathway^27–29^. A recent study on atherosclerotic mouse models (males only) demonstrated that downregulation of miR-133b inhibited the Notch-signaling pathway through elevated protein expression of Mastermind-like 1 and subsequently improved the atherosclerotic pathology by, among other, reducing the area of vulnerable plaques^29^. Interestingly, they also demonstrated that a downregulation of miR-133b and inhibition of the Notch-signaling pathway suppressed macrophage proliferation and migration and promoted macrophage apoptosis. Even though the link between miR-133b, Mastermind-like 1 and macrophages during the different stages of atherosclerosis need to be further explored, this study support our findings, suggesting that miR-133b is related to coronary plaques and that increasing concentrations has unfavorable effects on coronary plaque vulnerability.miRs have been explored as possible new and effective prognostic and diagnostic biomarkers of both acute and chronic CVDs^10,30–37^. For instance, miRs are explored as an addition to troponin in diagnosing of ST-segment elevation MI^38^. miR-133a and miR-133b have both been showed to increase shortly after myocardial ischemia, even before the peak of troponin levels^39^. miR-133a and miR-133b are transcribed by different loci but have a near identical mature sequence, differentiating only in one base at the 3’-terminal. miR-133a is known to be cardiac-specific and important in cardiac pathology, and an increased concentration of miR-133a during myocardial ischemia indicates cardiac damage^40^. On the other hand, miR-133b is not known to be highly present in cardiac cells but are, among other, involved in the regulation of atherosclerotic plaque towards instability and rupture^41^. In the present study, we found an association between increased concentration of miR-133b and increased odds of lipid-rich coronary plaques and no association between miR-133a and lipid-rich coronary plaques. Therefore, the increased concentration of miR-133b during myocardial ischemia may be more related to the destabilization of plaques rather than an expression of cardiac damage. Overall, it should be noted that miR-133b in CVD are not extensively studied and the existing studies demonstrate conflicting findings. In contrast to our findings, Kumar, et al.^34^ found decreased concentration of miR-133b with increasing severity of CAD.

Only a few studies have previously investigated the association between circulating miRs and vulnerable coronary plaque characteristics measured with the use of advanced invasive imaging techniques, such as NIRS, IVUS and/or optical coherence tomography (OCT)^13–18^. These studies were limited by the inclusion of few predefined candidate miRs with 21 analyzed miRs in total, and miR-133b was not among the analyzed miRs. One recent IVUS study found six circulating plasma miRs (miR-15a-5p, miR-30e-5p, miR-92a-3p, miR-199a-3p, miR-221-3p, and miR-222-3p) to be associated with coronary plaque necrotic core volume, and three miRs (miR-15a-5p, miR-93-5p, and miR-451a) to be associated with observed regression of plaque burden after 12 weeks of aerobic exercise^17^. Interestingly, miR-15a-5p, miR-30e-5p, and miR-199a-3p were found be involved in regulation of atherosclerosis-related pathways, including fatty acid biosynthesis and metabolism. Of all the miRs detected by Taraldsen, et al.^17^, only miR-92a was analyzed by others^16,18^. However, these could not demonstrate a correlation between miR-92a and any of the coronary plaque characteristics measured with OCT and IVUS, respectively. Studies have demonstrated that miRs can affect cells and different pathways associated with coronary atherosclerosis that may influence the plaques’ vulnerable features^13,42,43^. We present miR-133b as a potential new biomarker of lipid content in coronary plaques, a vulnerable plaque feature known to increase the risk of future CVD related events^6^. To the best of our knowledge, no other study has investigated the association between miR-133b and lipid-rich coronary plaques or other vulnerable coronary plaque characteristics.

An evident limitation in the present study is the small sample size and the large number of analyzed miRs. However, a broad search of miRs allows for the detection of potential important miRs associated with lipid-rich coronary plaques that may be missed by selecting candidate miRs. Furthermore, it was the single most diseased non-culprit lesion that was targeted in the present study, and the overall disease status of the coronary tree was not considered. This may have influenced the concentration of miRs in the plasma, together with vessel trauma induced by PCI, and potentially also statin therapy. The YELLOW trial detected changes in lipid content after short and intensive statin therapy versus standard statin therapy^44^. The CENIT trial approached this by including patients on stable statin therapy, with unchanged statin type and dosage, starting at least 6 weeks prior to inclusion^19^. A strength in the present study is the advanced coronary imaging technology, and data interpretation performed by an independent core facility. Lastly, sex-differences exists in various CVDs, and this may also apply for miR concentrations^35,45,46^. Sex-differences may explain the poor reproducibility between existing studies, together with many other factors such as type of sample (plasma or serum), sample quality, normalization method, ethnicity, etc. For a possible clinical application of miRs, a standardized protocol and a sex-specific approach is necessary. In the present study, the association between miR-133b and lipid-rich coronary plaques was found in a predominantly male population. This must be taken into consideration when interpreting our results.

## Conclusion

In the present study, we found that miR-133b was the miR most strongly associated with lipid-rich coronary plaques in patients with stable CAD. The odds of lipid-rich coronary plaques increased with increasing concentration of miR-133b. Even though the evidence for an association was modest, our study supports recent evidence suggesting unfavorable effects of increased concentration of miR-133b in coronary atherosclerosis. Thus, miR-133b could be a potential circulating biomarker of lipid-rich coronary plaques and risk of future MI. However, the prognostic value and clinical relevance of miR-133b needs to be assessed in larger cohorts.

## Supplementary Information

Supplementary Information.

## Data Availability

All miR data quantified by qPCR and the dataset used in statistical analyses of the present study are available from the corresponding author on reasonable request.

## Abbreviations

MI: Myocardial infarction
miR/miRs: MicroRNA/microRNAs
CAD: Coronary artery disease
maxLCBI_4mm_: Maximum lipid core burden index within any 4 mm segment across the entire lesion
LCBI: Lipid core burden index
NIRS: Near infrared spectroscopy
CVD: Cardiovascular disease
CENIT: Impact of cardiac exercise training on lipid content in coronary atheromatous plaques evaluated by near-infrared spectroscopy trial
PCI: Percutaneous coronary intervention
IVUS: Intravascular ultrasound
LDL-C: Low-density lipoprotein cholesterol
HDL-C: High-density lipoprotein cholesterol
qPCR: Quantitative polymerase chain reaction
RT: Reverse transcription
cDNA: Complementary DNA
Cq: Quantification cycle
OR: Odds ratio
ROC: Receiver operating characteristic
AUC: Area under the curve
OCT: Optical coherence tomography

## References

[1] Ojha, N. & Dhamoon, A.S. Myocardial Infarction. in *StatPearls* (StatPearls Publishing, Treasure Island (FL), 2022).

[2] Schuurman AS (2018). Near-infrared spectroscopy-derived lipid core burden index predicts adverse cardiovascular outcome in patients with coronary artery disease during long-term follow-up. Eur. Heart J..

[3] Karlsson S (2019). Intracoronary near-infrared spectroscopy and the risk of future cardiovascular events. Open Heart.

[4] Madder RD (2016). Large lipid-rich coronary plaques detected by near-infrared spectroscopy at non-stented sites in the target artery identify patients likely to experience future major adverse cardiovascular events. Eur. Heart J. Cardiovasc. Imaging.

[5] Waksman R (2019). Identification of patients and plaques vulnerable to future coronary events with near-infrared spectroscopy intravascular ultrasound imaging: A prospective, cohort study. The Lancet.

[6] Erlinge D (2021). Identification of vulnerable plaques and patients by intracoronary near-infrared spectroscopy and ultrasound (PROSPECT II): A prospective natural history study. Lancet.

[7] Waxman S (2009). In vivo validation of a catheter-based near-infrared spectroscopy system for detection of lipid core coronary plaques: Initial results of the SPECTACL study. JACC Cardiovasc. Imaging.

[8] Friedman RC, Farh KK, Burge CB, Bartel DP (2009). Most mammalian mRNAs are conserved targets of microRNAs. Genome Res..

[9] Andreou I, Sun X, Stone PH, Edelman ER, Feinberg MW (2015). miRNAs in atherosclerotic plaque initiation, progression, and rupture. Trends Mol. Med..

[10] Viereck J, Thum T (2017). Circulating noncoding RNAs as biomarkers of cardiovascular disease and injury. Circ. Res..

[11] Kalayinia S, Arjmand F, Maleki M, Malakootian M, Singh CP (2021). MicroRNAs: Roles in cardiovascular development and disease. Cardiovasc. Pathol..

[12] Wronska A (2023). The role of microRNA in the development, diagnosis, and treatment of cardiovascular disease: Recent developments. J. Pharmacol. Exp. Ther..

[13] He W (2019). The relationship of MicroRNA-21 and plaque stability in acute coronary syndrome. Medicine (Baltimore).

[14] Zhu GF (2019). Inflammation-related microRNAs are associated with plaque stability calculated by IVUS in coronary heart disease patients. J. Interv. Cardiol..

[15] Knoka E (2020). Circulating plasma microRNA-126, microRNA-145, and microRNA-155 and their association with atherosclerotic plaque characteristics. J. Clin. Transl. Res..

[16] Leistner DM (2016). Transcoronary gradients of vascular miRNAs and coronary atherosclerotic plaque characteristics. Eur. Heart J..

[17] Taraldsen MD, Wiseth R, Videm V, Bye A, Madssen E (2022). Associations between circulating microRNAs and coronary plaque characteristics: Potential impact from physical exercise. Physiol. Genom..

[18] Soeki T (2015). Plasma microRNA-100 is associated with coronary plaque vulnerability. Circ. J..

[19] Vesterbekkmo EK (2022). CENIT (impact of cardiac exercise training on lipid content in coronary atheromatous plaques evaluated by near-infrared spectroscopy): a randomized trial. J. Am. Heart Assoc..

[20] Gardner CM (2008). Detection of lipid core coronary plaques in autopsy specimens with a novel catheter-based near-infrared spectroscopy system. JACC Cardiovasc. Imaging.

[21] Andersen CL, Jensen JL, Ørntoft TF (2004). Normalization of real-time quantitative reverse transcription-PCR data: A model-based variance estimation approach to identify genes suited for normalization, applied to bladder and colon cancer data sets. Cancer Res..

[22] R: A language and environment for statistical computing (R Core Team R Foundation for Statistical Computing, Vienna, Austria, 2022).

[23] Friedman J, Hastie T, Tibshirani R (2010). Regularization paths for generalized linear models via coordinate descent. J. Stat. Softw..

[24] Libby P (2021). The changing landscape of atherosclerosis. Nature.

[25] Wang Y, Wang Q, Xu D (2022). New insights into macrophage subsets in atherosclerosis. J. Mol. Med. (Berl.).

[26] Mitchelson KR, Qin WY (2015). Roles of the canonical myomiRs miR-1, -133 and -206 in cell development and disease. World J. Biol. Chem..

[27] Marracino L (2021). Adding a "notch" to cardiovascular disease therapeutics: a microRNA-based approach. Front. Cell Dev. Biol..

[28] Kumar D, Narang R, Saluja D, Srivastava K (2022). Functional association of miR-133b and miR-21 through novel gene targets ATG5, LRP6 and SGPP1 in coronary artery disease. Mol. Diagn. Ther..

[29] Zheng CG (2019). miR-133b downregulation reduces vulnerable plaque formation in mice with AS through inhibiting macrophage immune responses. Mol. Ther. Nucleic Acids.

[30] Pedersen, O. B.,* et al.* Expression of microRNA predicts cardiovascular events in patients with stable coronary artery disease. *Thromb Haemost* (2023). 10.1055/s-0042-1760258 36603835

[31] Cortez-Dias N (2016). Circulating miR-122-5p/miR-133b ratio is a specific early prognostic biomarker in acute myocardial infarction. Circ. J..

[32] Guo JF (2018). Association between elevated plasma microRNA-223 content and severity of coronary heart disease. Scand. J. Clin. Lab Investig..

[33] Rizzacasa B (2019). MiR-423 is differentially expressed in patients with stable and unstable coronary artery disease: A pilot study. PLoS ONE.

[34] Kumar D (2020). Circulatory miR-133b and miR-21 as novel biomarkers in early prediction and diagnosis of coronary artery disease. Genes (Basel).

[35] Bye A (2016). Circulating microRNAs predict future fatal myocardial infarction in healthy individuals—The HUNT study. J. Mol. Cell Cardiol..

[36] Velle-Forbord T (2019). Circulating microRNAs as predictive biomarkers of myocardial infarction: Evidence from the HUNT study. Atherosclerosis.

[37] Gigante B (2020). MicroRNA signatures predict early major coronary events in middle-aged men and women. Cell Death Dis..

[38] Scărlătescu AI, Micheu MM, Popa-Fotea NM, Dorobanțu M (2021). MicroRNAs in acute ST elevation myocardial infarction-a new tool for diagnosis and prognosis: Therapeutic implications. Int. J. Mol. Sci..

[39] D'Alessandra Y (2010). Circulating microRNAs are new and sensitive biomarkers of myocardial infarction. Eur. Heart J..

[40] Kuwabara Y (2011). Increased microRNA-1 and microRNA-133a levels in serum of patients with cardiovascular disease indicate myocardial damage. Circ. Cardiovasc. Genet..

[41] Cipollone F (2011). A unique microRNA signature associated with plaque instability in humans. Stroke.

[42] Singh S (2020). MiR-223-3p and miR-122-5p as circulating biomarkers for plaque instability. Open Heart.

[43] Sun D, Ma T, Zhang Y, Zhang F, Cui B (2021). Overexpressed miR-335-5p reduces atherosclerotic vulnerable plaque formation in acute coronary syndrome. J. Clin. Lab Anal..

[44] Kini AS (2013). Changes in plaque lipid content after short-term intensive versus standard statin therapy: The YELLOW trial (reduction in yellow plaque by aggressive lipid-lowering therapy). J. Am. Coll. Cardiol..

[45] Tsuji M (2017). Sexual dimorphisms of mRNA and miRNA in human/murine heart disease. PLoS ONE.

[46] Klinge CM (2015). miRNAs regulated by estrogens, tamoxifen, and endocrine disruptors and their downstream gene targets. Mol. Cell Endocrinol..

