# Supplementary material for: Associations between circulating microRNAs and lipid-rich coronary plaques measured with near-infrared spectroscopy
Source: Sci Rep. 2023 May 10;13:7580. doi: 10.1038/s41598-023-34642-6 (PMC10172303; doi:10.1038/s41598-023-34642-6)
Supplement: Supplementary file 1 — Supplementary Information. [file 41598_2023_34642_MOESM1_ESM.docx]

# Supplementary Information

**Supplementary Figure S1:** Flowchart of the enrollment.

**Supplementary Figure S2:** Distribution of all maxLCBI_4mm_ measurements among patients with and without lipid-rich plaques (maxLCBI_4mm_ ≥324.7).

**Supplementary Figure S3:** Heatmap illustrating the dependence between the presented miRs, as calculated by Spearman correlation coefficients.

**Supplementary Figure S4:** Receiver operator characteristic curve analyses for miR-133b and traditional lipid measurements.

**Supplementary Table S1:** Estimated odds ratios for lipid-rich plaques (maxLCBI_4mm_ ≥324.7) from elastic net and the percentage inclusion in the 1000 bootstrap samples for all analyzed miRs and CVD risk factors.

Supplementary Figure S1. Flowchart of the enrollment.


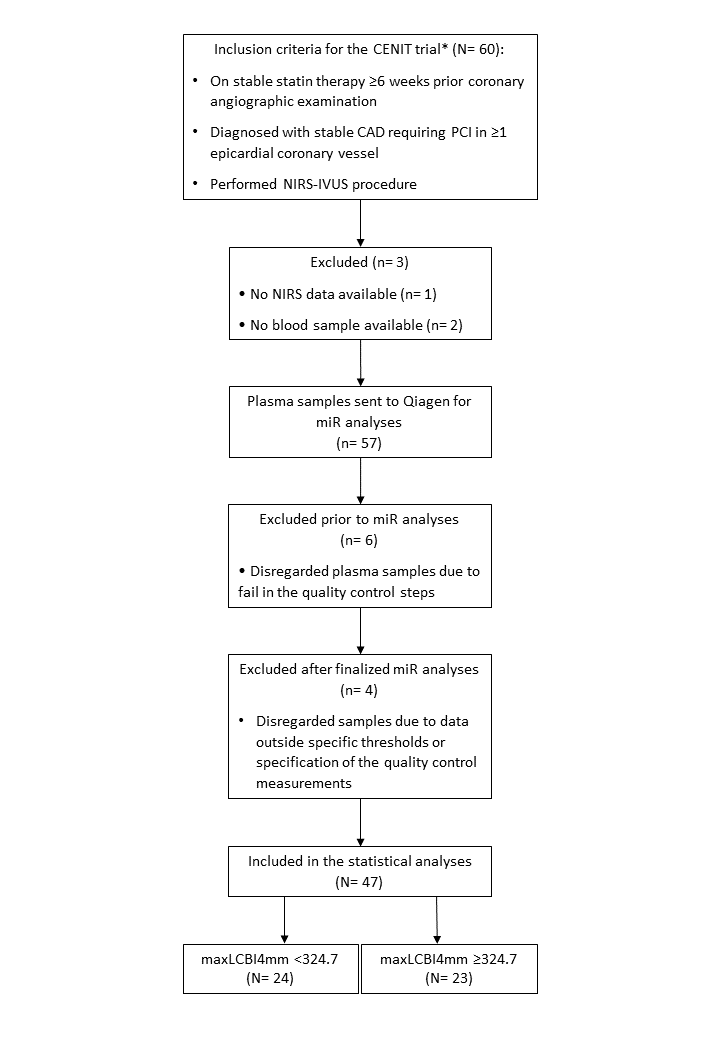


**Vesterbekkmo, E. K., Madssen, E., Aamot Aksetøy, I. L., Follestad, T., Nilsen, H. O., Hegbom, K.,Wisløff, U., & Wiseth, R. (2022). CENIT (Impact of Cardiac Exercise Training on Lipid Content in Coronary Atheromatous Plaques Evaluated by Near-Infrared Spectroscopy): A Randomized Trial. J Am Heart Assoc, 11(10), e024705.* [*https://doi.org/10.1161/jaha.121.024705*](https://doi.org/10.1161/jaha.121.024705)*.* CAD, coronary artery disease; PCI, percutaneous coronary intervention; NIRS-IVUS, near-infrared spectroscopy intravascular ultrasound; miR, microRNA; maxLCBI_4mm_, maximum lipid core burden index within any 4 mm segment across the entire lesion.

Supplementary Figure S2. Distribution of all maxLCBI_4mm_ measurements among patients with and without lipid-rich plaques (maxLCBI_4mm_ ≥324.7).


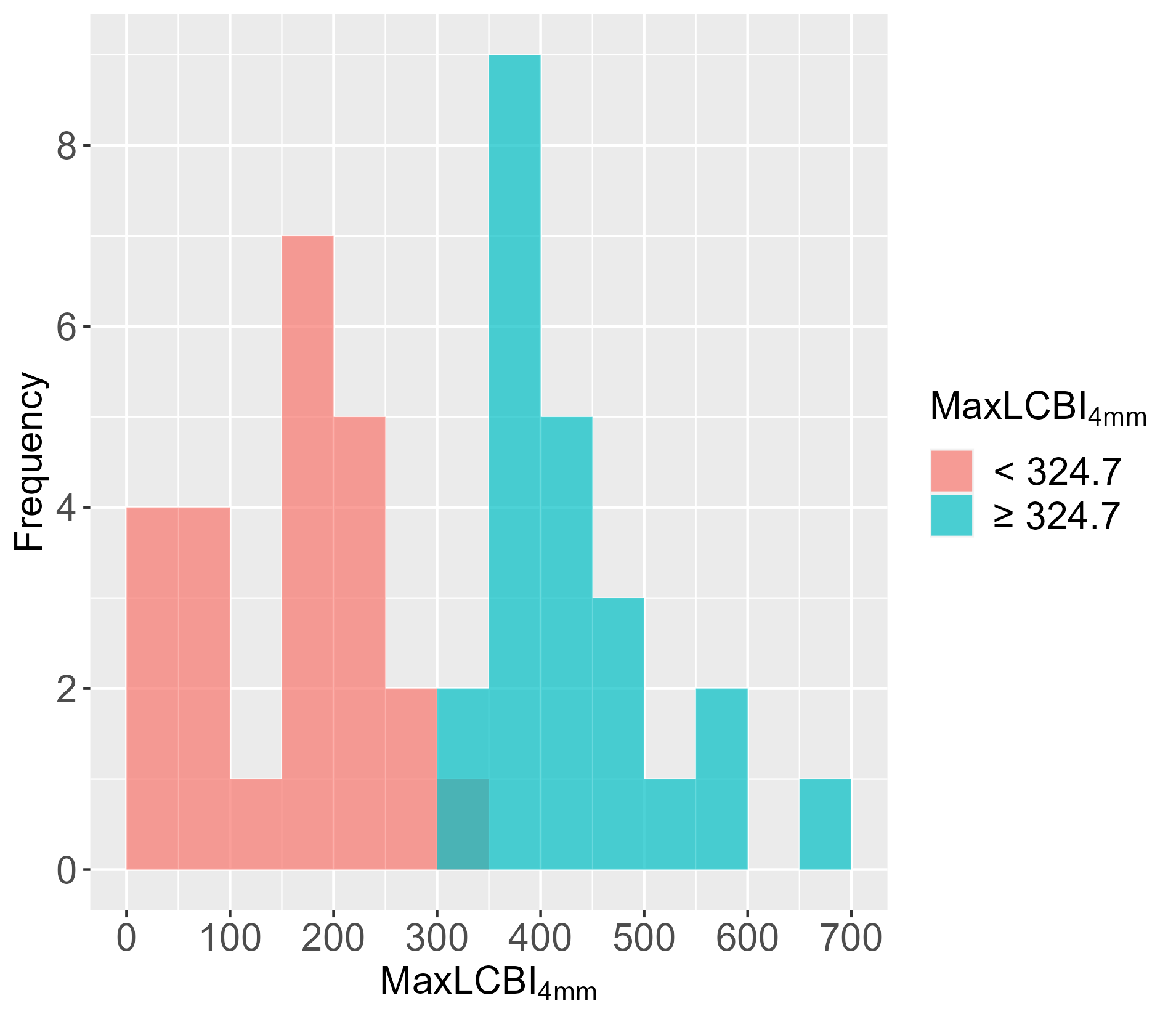


Histogram illustrating the distribution of all measured maxLCBI_4mm_ values in each of the two categories for the dichotomized maxLCBI_4mm_ variable. maxLCBI_4mm_, the maximum lipid core burden index within any 4 mm segment across the entire lesion.

Supplementary Figure S3. Heatmap illustrating the dependence between the presented miRs, as calculated by Spearman correlation coefficients.


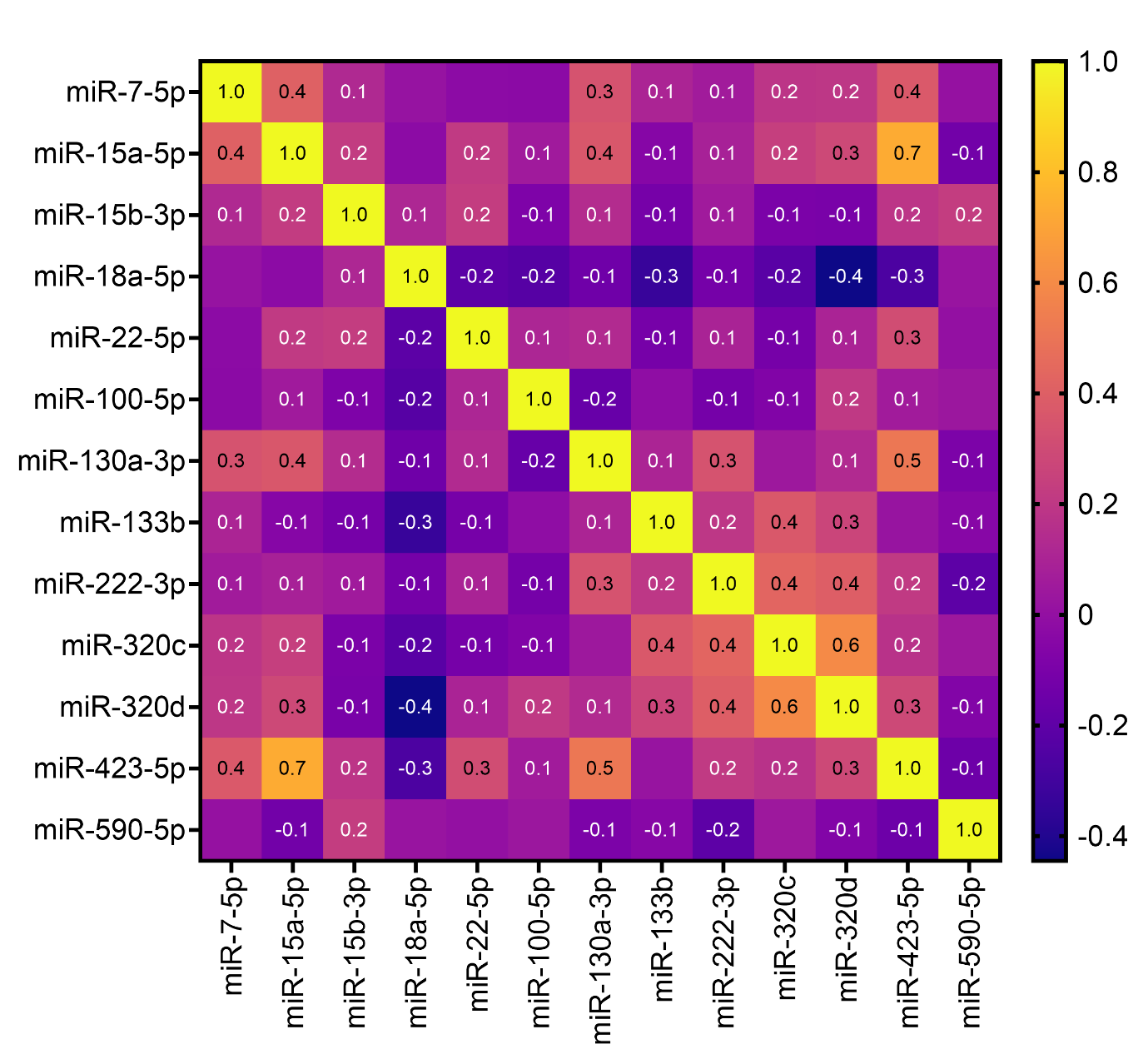


Heatmap illustrating the dependence between the miRs presented in the result section of the manuscript, showing Spearman correlation coefficients from -0.4 to 1. A correlation of ± 1 indicates perfect association, and 0 indicates no association. The strength of the correlation between the selected miRs are illustrated by colors, as described in the vertical column to the far right. The specific correlation coefficient for each comparison is also presented as numbers within each square. miR, microRNA; 3p, 3' arm of the precursor duplex; 5p, 5' arm of the precursor duplex.

Supplementary Figure S4. Receiver operator characteristic curve analyses for miR-133b and traditional lipid measurements.


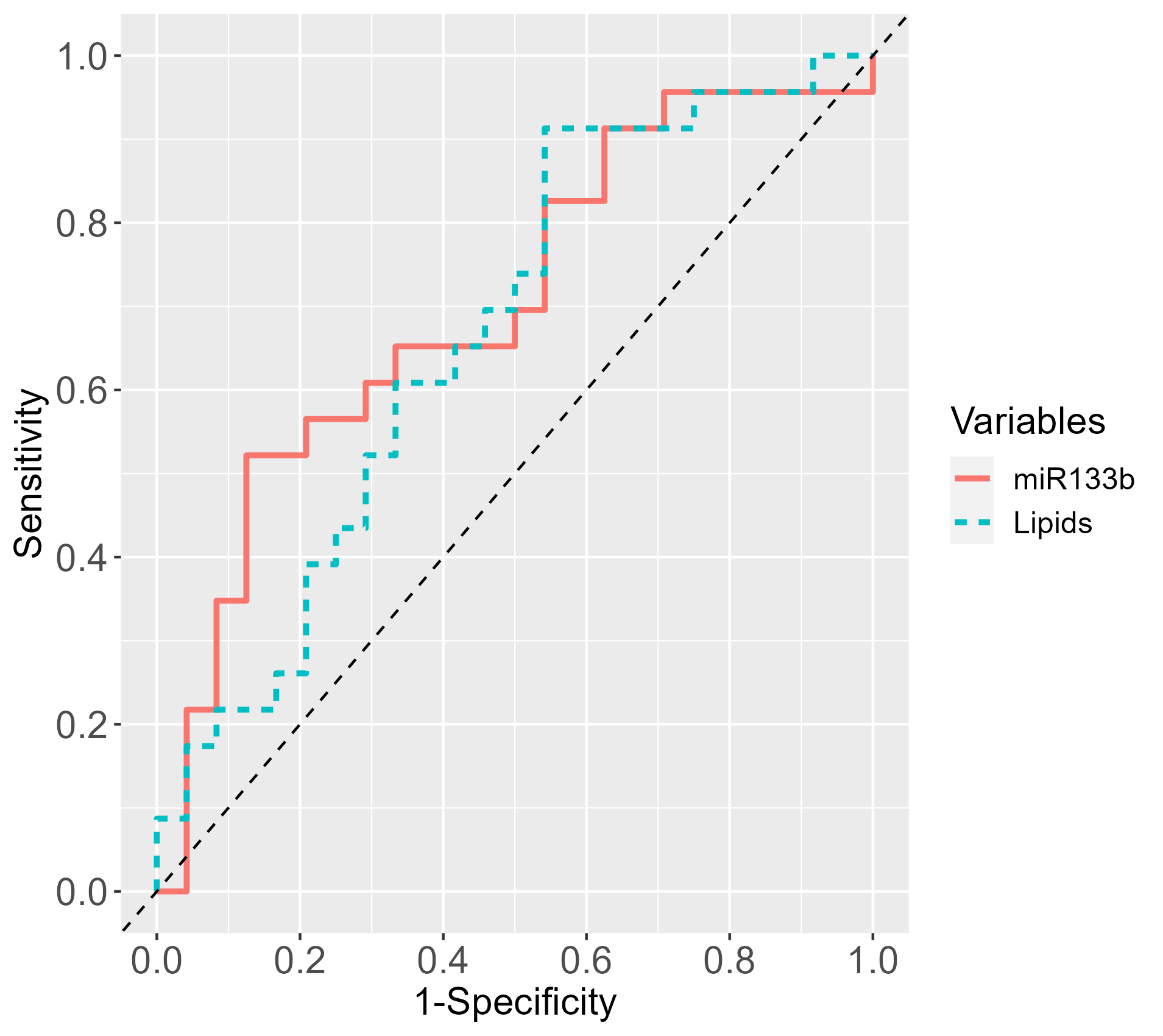
ROC curve analyses illustrating the predictive performance of miR-133b and traditional lipid measurements, including LDL cholesterol, HDL cholesterol, total triglycerides, LDL/HDL, and lipoprotein (a). The ROC curve and the area under the curve were calculated by cross-validation based on a logistic regression model with miR-133b as the only variable, and for a model with traditional lipid measurements. ROC, Receiver operating characteristic; LDL, low-density lipoprotein; HDL, high-density lipoprotein; miR, microRNA.

Supplementary Table S1. Estimated odds ratios for lipid-rich plaques (maxLCBI_4mm_ ≥324.7) from elastic net and the percentage presence in the 1000 bootstrap samples for the model including all analyzed miRs, and the model including miRs and cardiovascular disease risk factors.

|  | **Estimated odds ratio** | **Percentage presence in samples from bootstrap** |
| --- | --- | --- |
| **miRs only** | | |
| let-7a-5p | 1 | 15.5 |
| let-7b-3p | 1 | 8.8 |
| let-7b-5p | 1 | 0.6 |
| let-7c-5p | 1 | 8.0 |
| let-7d-3p | 1 | 3.0 |
| let-7d-5p | 1 | 2.2 |
| let-7e-5p | 1 | 1.7 |
| let-7f-5p | 1 | 2.7 |
| let-7g-5p | 1 | 9.1 |
| let-7i-5p | 1 | 2.3 |
| miR-100-5p | 1 | 35.0 |
| miR-101-3p | 1 | 14.3 |
| miR-103a-3p | 1 | 6.1 |
| miR-106a-5p | 1 | 0.2 |
| miR-106b-5p | 1 | 0.6 |
| miR-107 | 1 | 11.1 |
| miR-10b-5p | 1 | 3.1 |
| miR-122-5p | 1 | 0.6 |
| miR-125b-5p | 1 | 4.2 |
| miR-1260a | 1 | 8.1 |
| miR-126-3p | 1 | 16.5 |
| miR-126-5p | 1 | 3.1 |
| miR-128-3p | 1 | 2.9 |
| miR-130a-3p | 1 | 29 |
| miR-130b-3p | 1 | 3.3 |
| miR-132-3p | 1 | 11.5 |
| miR-133a-3p | 1 | 3.5 |
| miR-133b | 1.18 | 82.6 |
| miR-139-5p | 1 | 10.3 |
| miR-140-3p | 1 | 4.9 |
| miR-140-5p | 1 | 5.4 |
| miR-142-3p | 1 | 10.9 |
| miR-142-5p | 1 | 1.6 |
| miR-143-3p | 1 | 7.6 |
| miR-144-3p | 1 | 0.1 |
| miR-144-5p | 1 | 0.9 |
| miR-145-5p | 1 | 6.5 |
| miR-146a-5p | 1 | 1.6 |
| miR-146b-5p | 1 | 13.0 |
| miR-148a-3p | 1 | 5.3 |
| miR-148b-3p | 1 | 6.3 |
| miR-150-5p | 1 | 21.9 |
| miR-151a-3p | 1 | 0.3 |
| miR-151a-5p | 1 | 12.4 |
| miR-152-3p | 1 | 3.8 |
| miR-155-5p | 1 | 3.3 |
| miR-15a-5p | 1 | 26.5 |
| miR-15b-3p | 1 | 25.2 |
| miR-15b-5p | 1 | 1.5 |
| miR-162-3p | 1 | 3.5 |
| miR-16-5p | 1 | 1.0 |
| miR-17-5p | 1 | 2.4 |
| miR-181a-5p | 1 | 2.5 |
| miR-185-5p | 1 | 0.6 |
| miR-186-5p | 1 | 2.6 |
| miR-18a-5p | 1 | 35.6 |
| miR-18b-5p | 1 | 11.9 |
| miR-192-5p | 1 | 9.9 |
| miR-193a-5p | 1 | 11.9 |
| miR-194-5p | 1 | 2.6 |
| miR-195-5p | 1 | 6.2 |
| miR-197-3p | 1 | 13.6 |
| miR-199a-3p | 1 | 2.0 |
| miR-199a-5p | 1 | 4.5 |
| miR-19a-3p | 1 | 5.5 |
| miR-19b-3p | 1 | 0.1 |
| miR-200c-3p | 1 | 7.5 |
| miR-20a-5p | 1 | 0.0 |
| miR-20b-5p | 1 | 3.4 |
| miR-210-3p | 1 | 10.6 |
| miR-2110 | 1 | 7.2 |
| miR-215-5p | 1 | 10.9 |
| miR-21-5p | 1 | 16.9 |
| miR-221-3p | 1 | 5.3 |
| miR-222-3p | 1 | 27.5 |
| miR-223-3p | 1 | 1.3 |
| miR-223-5p | 1 | 5.9 |
| miR-22-3p | 1 | 2.0 |
| miR-22-5p | 1 | 28.4 |
| miR-23a-3p | 1 | 2.4 |
| miR-23b-3p | 1 | 0.2 |
| miR-24-3p | 1 | 2.1 |
| miR-25-3p | 1 | 0.8 |
| miR-26a-5p | 1 | 5.1 |
| miR-26b-5p | 1 | 0.2 |
| miR-27a-3p | 1 | 2.5 |
| miR-27b-3p | 1 | 0.8 |
| miR-28-3p | 1 | 2.2 |
| miR-28-5p | 1 | 2.0 |
| miR-29a-3p | 1 | 0.8 |
| miR-29b-3p | 1 | 5.8 |
| miR-29c-3p | 1 | 21.2 |
| miR-301a-3p | 1 | 6.0 |
| miR-30a-5p | 1 | 5.7 |
| miR-30b-5p | 1 | 4.2 |
| miR-30c-5p | 1 | 8.4 |
| miR-30d-5p | 1 | 4.1 |
| miR-30e-3p | 1 | 3.9 |
| miR-30e-5p | 1 | 8.1 |
| miR-320a | 1 | 2.8 |
| miR-320b | 1 | 3.6 |
| miR-320c | 1 | 9.7 |
| miR-320d | 1 | 32.8 |
| miR-324-3p | 1 | 5.1 |
| miR-324-5p | 1 | 5.3 |
| miR-32-5p | 1 | 3.8 |
| miR-328-3p | 1 | 2.9 |
| miR-331-3p | 1 | 5.7 |
| miR-335-3p | 1 | 11.8 |
| miR-335-5p | 1 | 2.9 |
| miR-338-3p | 1 | 9.4 |
| miR-339-3p | 1 | 20.1 |
| miR-339-5p | 1 | 3.4 |
| miR-33a-5p | 1 | 5.2 |
| miR-342-3p | 1 | 5.1 |
| miR-34a-5p | 1 | 5.1 |
| miR-361-5p | 1 | 1.9 |
| miR-363-3p | 1 | 6.9 |
| miR-365a-3p | 1 | 6.0 |
| miR-374a-5p | 1 | 0.6 |
| miR-374b-5p | 1 | 1.3 |
| miR-375 | 1 | 21.1 |
| miR-376a-3p | 1 | 1.7 |
| miR-376c-3p | 1 | 3.2 |
| miR-409-3p | 1 | 19.3 |
| miR-421 | 1 | 6.7 |
| miR-423-3p | 1 | 2.0 |
| miR-423-5p | 1 | 21.3 |
| miR-424-5p | 1 | 25.7 |
| miR-425-3p | 1 | 7.2 |
| miR-425-5p | 1 | 6.5 |
| miR-451a | 1 | 6.6 |
| miR-454-3p | 1 | 0.4 |
| miR-483-5p | 1 | 6.7 |
| miR-484 | 1 | 6.3 |
| miR-486-5p | 1 | 4.8 |
| miR-495-3p | 1 | 3.8 |
| miR-497-5p | 1 | 2.3 |
| miR-501-3p | 1 | 8.4 |
| miR-502-3p | 1 | 11.6 |
| miR-505-3p | 1 | 2.2 |
| miR-532-3p | 1 | 7.6 |
| miR-574-3p | 1 | 14.0 |
| miR-584-5p | 1 | 18.7 |
| miR-590-5p | 1 | 28.2 |
| miR-629-5p | 1 | 10.4 |
| miR-652-3p | 1 | 2.1 |
| miR-660-5p | 1 | 18.8 |
| miR-71-3p | 1 | 13.7 |
| miR-7-5p | 1 | 31.2 |
| miR-766-3p | 1 | 12.2 |
| miR-874-3p | 1 | 14.0 |
| miR-877-5p | 1 | 25.4 |
| miR-885-5p | 1 | 2.4 |
| miR-92a-3p | 1 | 6.1 |
| miR-92b-3p | 1 | 8.7 |
| miR-93-3p | 1 | 15.3 |
| miR-93-5p | 1 | 0.7 |
| miR-99a-5p | 1 | 2.6 |
| miR-99b-5p | 1 | 5.4 |
| **miRs and established CVD risk factors** | | |
| Age | 1 | 1.7 |
| Medically treated hypertension | 1 | 25.0 |
| Hyperlipidemia | 1 | 9.5 |
| Diabetes mellitus | 1 | 9.7 |
| Previous CVD | 1 | 20.4 |
| Hereditary CVD | 1 | 54.9 |
| Body mass index | 1 | 1.1 |
| Current smoker or ex-smoker | 1 | 8.6 |
| Total cholesterol | 1 | 1.4 |
| HDL-C | 1 | 6.1 |
| LDL-C | 1 | 4.3 |
| Total triglycerides | 1 | 6.8 |
| LDL-C/HDL-C | 1 | 2.3 |
| Lipoprotein a | 1 | 10.7 |
| let-7a-5p | 1 | 17.7 |
| let-7b-3p | 1 | 8.1 |
| let-7b-5p | 1 | 0.6 |
| let-7c-5p | 1 | 8.6 |
| let-7d-3p | 1 | 3.8 |
| let-7d-5p | 1 | 1.9 |
| let-7e-5p | 1 | 2.1 |
| let-7f-5p | 1 | 2.5 |
| let-7g-5p | 1 | 4.0 |
| let-7i-5p | 1 | 1.8 |
| miR-100-5p | 1 | 31.9 |
| miR-101-3p | 1 | 16.4 |
| miR-103a-3p | 1 | 4.6 |
| miR-106a-5p | 1 | 0.1 |
| miR-106b-5p | 1 | 0.1 |
| miR-107 | 1 | 9.0 |
| miR-10b-5p | 1 | 2.7 |
| miR-122-5p | 1 | 0.4 |
| miR-125b-5p | 1 | 4.2 |
| miR-1260a | 1 | 5.0 |
| miR-126-3p | 1 | 15.1 |
| miR-126-5p | 1 | 2.2 |
| miR-128-3p | 1 | 3.6 |
| miR-130a-3p | 1 | 31.0 |
| miR-130b-3p | 1 | 2.6 |
| miR-132-3p | 1 | 13.4 |
| miR-133a-3p | 1 | 3.1 |
| miR-133b | 1.15 | 84.2 |
| miR-139-5p | 1 | 8.6 |
| miR-140-3p | 1 | 4.1 |
| miR-140-5p | 1 | 6.2 |
| miR-142-3p | 1 | 11.5 |
| miR-142-5p | 1 | 1.8 |
| miR-143-3p | 1 | 5.2 |
| miR-144-3p | 1 | 0.0 |
| miR-144-5p | 1 | 0.5 |
| miR-145-5p | 1 | 5.3 |
| miR-146a-5p | 1 | 2.0 |
| miR-146b-5p | 1 | 12.1 |
| miR-148a-3p | 1 | 4.0 |
| miR-148b-3p | 1 | 3.9 |
| miR-150-5p | 1 | 22.1 |
| miR-151a-3p | 1 | 0.1 |
| miR-151a-5p | 1 | 11.4 |
| miR-152-3p | 1 | 2.7 |
| miR-155-5p | 1 | 2.5 |
| miR-15a-5p | 1 | 25.3 |
| miR-15b-3p | 1 | 28.7 |
| miR-15b-5p | 1 | 1.4 |
| miR-162-3p | 1 | 4.1 |
| miR-16-5p | 1 | 0.8 |
| miR-17-5p | 1 | 1.3 |
| miR-181a-5p | 1 | 2.1 |
| miR-185-5p | 1 | 0.5 |
| miR-186-5p | 1 | 2.2 |
| miR-18a-5p | 1 | 33.3 |
| miR-18b-5p | 1 | 5.2 |
| miR-192-5p | 1 | 11.3 |
| miR-193a-5p | 1 | 7.3 |
| miR-194-5p | 1 | 2.1 |
| miR-195-5p | 1 | 4.8 |
| miR-197-3p | 1 | 9.8 |
| miR-199a-3p | 1 | 2.8 |
| miR-199a-5p | 1 | 3.7 |
| miR-19a-3p | 1 | 6.6 |
| miR-19b-3p | 1 | 0.0 |
| miR-200c-3p | 1 | 4.7 |
| miR-20a-5p | 1 | 0.1 |
| miR-20b-5p | 1 | 2.7 |
| miR-210-3p | 1 | 9.3 |
| miR-2110 | 1 | 5.8 |
| miR-215-5p | 1 | 11.1 |
| miR-21-5p | 1 | 15.1 |
| miR-221-3p | 1 | 5.0 |
| miR-222-3p | 1 | 27.6 |
| miR-223-3p | 1 | 1.2 |
| miR-223-5p | 1 | 5.1 |
| miR-22-3p | 1 | 3.3 |
| miR-22-5p | 1 | 29.3 |
| miR-23a-3p | 1 | 2.1 |
| miR-23b-3p | 1 | 0.0 |
| miR-24-3p | 1 | 1.6 |
| miR-25-3p | 1 | 0.7 |
| miR-26a-5p | 1 | 4.4 |
| miR-26b-5p | 1 | 0.3 |
| miR-27a-3p | 1 | 2.3 |
| miR-27b-3p | 1 | 0.8 |
| miR-28-3p | 1 | 1.5 |
| miR-28-5p | 1 | 1.7 |
| miR-29a-3p | 1 | 1.6 |
| miR-29b-3p | 1 | 8.8 |
| miR-29c-3p | 1 | 17.6 |
| miR-301a-3p | 1 | 5.1 |
| miR-30a-5p | 1 | 4.0 |
| miR-30b-5p | 1 | 5.2 |
| miR-30c-5p | 1 | 8.5 |
| miR-30d-5p | 1 | 2.5 |
| miR-30e-3p | 1 | 1.0 |
| miR-30e-5p | 1 | 6.5 |
| miR-320a | 1 | 3.1 |
| miR-320b | 1 | 4.7 |
| miR-320c | 1 | 8.3 |
| miR-320d | 1 | 32.8 |
| miR-324-3p | 1 | 4.7 |
| miR-324-5p | 1 | 4.6 |
| miR-32-5p | 1 | 4.0 |
| miR-328-3p | 1 | 2.4 |
| miR-331-3p | 1 | 6.3 |
| miR-335-3p | 1 | 9.2 |
| miR-335-5p | 1 | 2.7 |
| miR-338-3p | 1 | 9.1 |
| miR-339-3p | 1 | 19.0 |
| miR-339-5p | 1 | 1.8 |
| miR-33a-5p | 1 | 5.6 |
| miR-342-3p | 1 | 4.2 |
| miR-34a-5p | 1 | 4.6 |
| miR-361-5p | 1 | 1.3 |
| miR-363-3p | 1 | 4.6 |
| miR-365a-3p | 1 | 6.1 |
| miR-374a-5p | 1 | 0.8 |
| miR-374b-5p | 1 | 0.8 |
| miR-375 | 1 | 14.1 |
| miR-376a-3p | 1 | 1.8 |
| miR-376c-3p | 1 | 4.7 |
| miR-409-3p | 1 | 17.1 |
| miR-421 | 1 | 8.9 |
| miR-423-3p | 1 | 1.3 |
| miR-423-5p | 1 | 21.2 |
| miR-424-5p | 1 | 23.4 |
| miR-425-3p | 1 | 5.7 |
| miR-425-5p | 1 | 5.4 |
| miR-451a | 1 | 6.6 |
| miR-454-3p | 1 | 0.4 |
| miR-483-5p | 1 | 6.7 |
| miR-484 | 1 | 3.2 |
| miR-486-5p | 1 | 6.7 |
| miR-495-3p | 1 | 4.1 |
| miR-497-5p | 1 | 3.2 |
| miR-501-3p | 1 | 9.2 |
| miR-502-3p | 1 | 10.5 |
| miR-505-3p | 1 | 1.8 |
| miR-532-3p | 1 | 6.9 |
| miR-574-3p | 1 | 14.4 |
| miR-584-5p | 1 | 18.4 |
| miR-590-5p | 1 | 27.6 |
| miR-629-5p | 1 | 10.0 |
| miR-652-3p | 1 | 1.8 |
| miR-660-5p | 1 | 17.8 |
| miR-71-3p | 1 | 12.4 |
| miR-7-5p | 1 | 31.5 |
| miR-766-3p | 1 | 10.8 |
| miR-874-3p | 1 | 12.0 |
| miR-877-5p | 1 | 25.0 |
| miR-885-5p | 1 | 1.5 |
| miR-92a-3p | 1 | 8.0 |
| miR-92b-3p | 1 | 4.7 |
| miR-93-3p | 1 | 15.5 |
| miR-93-5p | 1 | 0.5 |
| miR-99a-5p | 1 | 2.0 |
| miR-99b-5p | 1 | 2.9 |

maxLCBI_4mm_, the maximum lipid core burden index within any 4 mm segment across the entire lesion; let, lethal; miR/miRs, microRNA(s); 3p, 3' arm of the precursor duplex; 5p, 5' arm of the precursor duplex; LDL-C, low-density lipoprotein cholesterol; HDL-C, high-density lipoprotein cholesterol; CVD, cardiovascular disease.
